# Prospects of Testing Diurnal Profiles of Expressions of TSH-R and Circadian Clock Genes in Thyrocytes for Identification of Preoperative Biomarkers for Thyroid Carcinoma

**DOI:** 10.3390/ijms232012208

**Published:** 2022-10-13

**Authors:** Arcady Putilov

**Affiliations:** 1 Research Group for Math-Modeling of Biomedical Systems, Research Institute for Molecular Biology and Biophysics of the Federal Research Centre for Fundamental and Translational Medicine, 630117 Novosibirsk, Russia; putilov@ngs.ru; Tel.: +49-30-53674643 or +49-30-61290031; 2 Laboratory of Sleep/Wake Neurobiology, Institute of Higher Nervous Activity and Neurophysiology of the Russian Academy of Sciences, 117865 Moscow, Russia; 3 Laboratory of Nanobiotechnology and Biophysics, North-Caucasus Federal University, 355029 Stavropol, Russia

**Keywords:** thyroid cancer, thyroid nodules, circadian clockwork, TSH, TSH-R, circadian clock genes

## Abstract

Thyroid Nodules (TN) are frequent but mostly benign, and postoperative rate of benign TN attains the values from 70% to 90%. Therefore, there is an urgent need for identification of reliable preoperative diagnosis markers for patients with indeterminate thyroid cytology. In this study, an earlier unexplored design of research on preoperative biomarkers for thyroid malignancies was proposed. Evaluation of reported results of studies addressing the links of thyroid cancer to the circadian clockwork dysfunctions and abnormal activities of Thyroid-Stimulating Hormone (TSH) and its receptor (TSH-R) suggested diagnostic significance of such links. However, there is still a gap in studies of interrelationships between diurnal profiles of expression of circadian clock genes and TSH-R in indeterminate thyroid tissue exposed to different concentrations of TSH. These interrelationships might be investigated in future in vitro experiments on benign and malignant thyrocytes cultivated under normal and challenged TSH levels. Their design requires simultaneous measurement of diurnal profiles of expression of both circadian clock genes and TSH-R. Experimental results might help to bridge previous studies of preoperative biomarkers for thyroid carcinoma exploring diagnostic value of diurnal profiles of serum TSH levels, expression of TSH-R, and expression of circadian clock genes.

## 1. Introduction

Thyroid cancer is a common endocrine malignancy that accounts for about 1% of all human malignancies [1,2,3]. It usually presents as Thyroid Nodules (TN) defined as any discrete mass in the thyroid gland. Although such nodules are frequent, most of them are benign and only about 5% are cancerous [4,5]. Such a low rate of cancerous TN suggests the risks of unnecessary surgeries for asymptomatic benign TN and delays of diagnostic and treatment of asymptomatic cancerous TN. Therefore, it is crucial to establish an adequate differential preoperative diagnosis for thyroid carcinoma [6].

There are five main histological types of thyroid cancers: papillary, follicular, poorly differentiated, undifferentiated/anaplastic (this is the most aggressive form), and medullary thyroid cells (unlike other types, they arise from neuroendocrine C cells) [7]. Papillary and follicular types are considered to be differentiated. The majority of thyroid tumors are classified as well differentiated (85%) [8]. The prognosis of patients with differentiated type cancer is optimistic because they are usually curable with surgery and radioactive iodine therapy [9,10,11]. Poorly differentiated carcinomas are characterized by an incomplete tumor capsule with expansive growth. Only a small proportion of thyroid tumors (about 5–7%) lose their features of cell origin and they are classified as either poorly differentiated or undifferentiated/anaplastic [8]. The undifferentiated form has no chance of cure, and it is associated with rapid progression and a high risk of mortality [12].

Routine diagnosis of thyroid nodules usually relies on Fine Needle Aspiration (FNA) biopsy, which served until recently as the most accurate and safe tool for clinical evaluation of non-secreting TN [5]. FNA improves the quality of life for patients with TN by allowing prevention of unnecessary surgery in patients with benign lesions and provides better managing of malignancies in patients with the lesions of undetermined significance and the lesions diagnosed as suspicious for a follicular neoplasm [13,14]. Surgery is recommended only after the evaluation of the FNA results aimed on identification of thyroid malignancies. Despite the applying of FNA, a high rate of unnecessary surgery was revealed. Postoperatively, 70–90% of thyroid FNA cases are benign [15]. For instance, the distinction between benign and malignant nodules based on cytological features is impossible for approximately 30% of cases that are lacking the morphological features needed for providing definitive classification [14,16,17].

Due to the difficulties of distinguishing benign TN from malignant non-functional TN, the diagnostic approach to thyroid cancer has become one of the most challenging issues in oncology of the thyroid system [18]. The development of preoperative markers for thyroid carcinoma plays a critical role in the efforts to reduce the rate of unnecessary surgery. Despite intensive research aimed on uncovering reliable biomarkers for thyroid carcinoma (e.g., [19,20,21,22,23,24,25,26,27]), the examinations of various previously proposed immunohistochemical and genetic markers of thyroid malignancy led to the conclusion that many of them failed to provide accurate distinguishing between follicular adenoma and carcinoma (e.g., [28]).

The diagnostic and management of cancer patients has been recently revolutionized by the introduction of liquid biopsy and molecular testing platforms [18,29,30,31,32,33,34,35,36,37]. Liquid biopsy provided a possibility of elimination of the invasive procedures needed to obtain tissue samples. It detects and analyzes biological samples released from the tumor into the bloodstream [18,29,30,31,32]. Therefore, such biopsy can be repeatedly performed in a noninvasive way, at lower cost and without the risks associated with classic tissue biopsy [32]. As a non-invasive approach for the detection of diagnostic biomarkers for early tumor diagnosis, prognosis, and disease monitoring, liquid biopsy appears to be more beneficial than FNA biopsy [18]. The molecular testing platforms present another recently introduced option for improving the presurgical diagnosis in indeterminate TN [33]. These molecular tests have been developed for reducing the avoidable treatment of benign TN and optimization of surgical management. Since these platforms are now frequently used as an integral part of the cytologic evaluation in conjunction with FNA to provide more definitive guidance for the decision making in clinic including the decision to avoid unnecessary surgical interventions [33,34,35,36,37]. 

The testing of molecular biomarkers for cancer patient stratification become mandatory in the recent years [18], and, therefore, it is critical to intensify research on preoperative biomarkers for thyroid carcinoma [37,38,39].

This perspective article was inspired by the findings pointing out the involvement of the circadian clockwork dysfunctions into malignant transformation of thyroid tissue (e.g., [39]) that are in line with one of the earlier findings suggesting the involvement of the clock-regulated thyroid hormone in thyroid cancer (e.g., [40]). These studies provided evidence for the associations of thyroid cancer with both these dysfunctions and abnormal characteristics of this hormone activity. Therefore, the present perspective article is arguing for the plausibility of aiming future studies of preoperative biomarkers for thyroid cancer on testing both the circadian clockwork and activity of this hormone receptor in benign and malignant thyrocytes. 

## 2. Results

Since the circadian clock machinery is of great importance for an organism, it is natural to expect that its abnormal functioning can serve as a specific marker for thyroid carcinoma. Evidence for the importance of the circadian clock machinery for most multi-cell organisms was proved by the discovery that each cell in almost each tissue of the organism contains a set of genes for generating its own circadian clocks with a near 24-h (circadian) period [41,42]. These so-called circadian clock genes also allow the establishment of circadian clocks with a similar molecular makeup at any of the higher (multicell) hierarchical levels, e.g., peripheral clocks of various tissues, organs, physiological and endocrine systems, and the central clocks of the whole organism [43,44]. The circadian clocks control the expression of almost half of the protein coding genes for coordinating diurnal rhythmicity of different biochemical, physiologic, and behavioral functions in the organism [45,46]. The reports suggesting the modulating influence of the cellular circadian clocks on gene expression, cell division, DNA repair, apoptosis control, inflammation, etc. (e.g., [47]) gave rise to the hypotheses that the dysfunction of these clocks may have such a serious pathological effect as cancer [48,49,50,51,52,53,54,55,56]. Indeed, accumulating fundings indicate that the disruptions of circadian rhythms are often linked to cancer [57,58,59,60,61,62,63]. Therefore, several research groups suggested a possibility to establish a relationship between thyroid cancer and circadian clockwork dysfunctions (e.g., [2,39,64,65]).

### 2.1. One of Hormonal Markers of the Circadian Clockwork in Thyroid Carcinoma

Among several hormones secreted by the thyroid gland, only thyrotropin (Thyroid-Stimulating Hormone or TSH) exhibits a clear diurnal rhythmicity of secretion, stimulates the thyroid gland to produce thyroxine (T3) and triiodothyronine (T4), but, in contrast, the concentrations of free T3 and T4 remain relatively stable throughout a day. Due to the diurnal variation in TSH concentration in blood, the 24-h profile of this hormone in serum serves as one of most robust markers of the circadian clockwork of the human organism [66,67,68,69,70]. It is known that the central clocks in the suprachiasmatic nuclei exert their influence on TSH via neuronal and humoral signals but the peripheral tissues containing similar circadian clock proteins are also involved in its regulation [71,72]. The disruptions of TSH levels in serum have been associated with shift work, jet lag, and chronic sleep disorders (see [70] for review). 

TSH plays a pivotal role in controlling the hypothalamic–pituitary–thyroid axis and serves as the most reliable physiologic marker of thyroid hormones activity [2]. Results of intensive testing of TSH concentrations in serum suggested the increase of the risk of malignancy in thyroid nodules in parallel with the increase of these concentrations [73]. The results of published studies suggested that the likelihood of thyroid cancer increased with increase of serum TSH concentrations [74], that patients with high serum TSH levels had a significantly higher risk of differentiated carcinoma than patients with low levels [75], that higher TSH concentrations, even within the normal range, were associated with a subsequent diagnosis of thyroid cancer in individuals with thyroid abnormalities [76], that the levels of serum TSH in the group with differentiated thyroid cancer was significantly increased compared with that in the benign TN group [77], and that TSH levels in patients of stage 3 were significantly higher than those in patients in stages 2 and 1 [78], etc. Therefore, it was concluded that levels of this hormone in serum might have a diagnostic value in the preoperational management of thyroid carcinoma [79,80,81,82,83,84,85,86]. 

However, it is important to note that, when serum TSH levels in a group with thyroid cancer were reported to be significantly higher than those in a benign TN group, they often remained within a normal range for healthy people. Due to the huge overlap and the small difference in median serum TSH levels between patients with benign and malignant TN, median serum TSH levels cannot be considered the biomarker of choice for thyroid cancer in a clinical setting [87]. Moreover, it was noted that preoperative concentrations of TSH in serum might be measured for prediction of risk of differentiated thyroid cancers, but they failed to be a good risk predictor at an early stage of this cancer development (in microcarcinomas) [88]. Therefore, it is necessary to conclude that the measurement of serum TSH levels represents an easily performed but only additional tool for decision-making in patients with indeterminate cytological findings [89,90,91].

Overall, the results suggesting significant association between thyroid cancer and increased TSH levels in serum [73,74,75,76,77,78,79,80,81,82,83,84,85,86] pointed at an important but not very high diagnostic value of these levels in the preoperative management of thyroid carcinoma [89,90,91]. 

Moreover, the dependency upon TSH levels was demonstrated for thyroid-cell proliferation, and an association between higher TSH levels and a postoperative diagnosis of differentiated thyroid cancer was reported. The results of such experimental studies were enriched by clinical observations thus providing the rationale for the implicating TSH suppression into postoperative management of thyroid function in differentiated thyroid cancer [92,93,94,95,96].

### 2.2. Expression of Circadian Clock Genes in Thyroid Carcinoma

In the mammalian cells, the circadian clocks are regulated by a transcriptional-translational feedback loop [97]. Firstly, the heterodimers are formed by BMAL1 and CLOCK or by BMAL1 and NPAS2. Secondly, these heterodimers activate the expression of CRY and PER (PER1, PER2, and PER3) genes. Thirdly, these genes are acting via E-box elements as transcription factors directed to the promoters of CRY and PER. Fourthly, that completes the feedback loop, and PER and CRY proteins form heterodimers suppressing activity of BMAL1/CLOCK or BMAL1/NPAS2. The circadian expression of BMAL1 and NPAS2 is additionally influenced by such nuclear receptors as RORα and REV-ERBα. They regulate the expression of BMAL1 and NPAS2 by targeting a ROR-response element in the promoters of BMAL1 and NPAS2 genes [97,98]. The function of these regulators is not limited to the control each other’s expression. They additionally drive rhythmic expression of thousands of target genes by binding cis-regulatory sites or through downstream transcriptional regulators. Circadian transcription factors also interact with a number of coactivators, corepressors, and chromatin-associated factors that read, write, or erase chromatin histone modification marks for activating or repressing transcription. The transcriptional activity of cellular circadian clocks enables a set of transcriptional regulators to temporally couple their activity with the synchronous rhythmic expression of thousands of genes with peak expression at distinct times of the day [99,100,101,102]. 

Accumulated fundings revealed some abnormalities in expression profiles of circadian clock genes in well-differentiated thyroid cancer but not in the benign TN [2,39,70,103]. For example, Mannic et al. [39] discovered the alternation of the circadian clock machinery in the thyroid tissue during malignant transformation. They found up-regulation of BMAL1 and the downregulation of CRY2 in tissues sampled from well-differentiated thyroid cancer. Importantly, they also observed the drastic changes in the expression of these circadian clock genes in poorly differentiated thyroid carcinoma [39]. 

It has to be noted that this study [39] provided the results of additional comparisons of the diurnal profiles of expression of several circadian clock genes in samples collected at different time of the day (i.e., during surgery performed in the time window between 8:00 AM and 2:00 PM) that were then synchronized in primary thyrocytes cultured in vitro for 7 days and harvested every 6 h during 36 h. It was found that the thyrocytes from healthy and benign TN perfectly kept their circadian properties (i.e., the endogenous clock gene expressions in these TN exhibited the circadian oscillatory patterns in synchronized thyrocytes). However, when these primary cultured thyrocytes were diagnosed as papillary and poorly differentiated thyroid carcinomas, the alternated circadian profiles were detected [39]. 

The results suggesting the upregulation of BMAL1 (ARNTL) and the downregulation of CRY2 [39] were supported by Lou et al. [64]. They found that, as compared to benign and healthy TN groups, malignant TN group showed higher expression levels of three circadian clock genes (CLOCK, BMAL1, and PER2) and lower expression levels of one such gene (CRY2). Significant alterations in the expression levels of BMAL1 were also confirmed by Chitikova et al. [63] in their study of patients with papillary thyroid carcinoma. Expression of BMAL1 (ARNTL) in TN was also tested by Sadowski et al. [103] in FNA samples obtained preoperatively from patients with papillary thyroid carcinoma. They found that in samples from these patients, as compared to samples of benign TN, expression of this circadian clock gene was upregulated [103].

Overall, these and several other studies provided evidence for a plausibility of improvement of preoperative diagnosis of thyroid cancer by testing the thyroid clock machinery in benign or malignant tissues [2,63,64,70,103,104,105].

### 2.3. One of the Thyroid-Specific Hormone Receptors in Thyroid Carcinoma

TSH directly bound to its receptor (TSH-R), and this receptor is mostly (but not exclusively) expressed in the thyroid cells [106]. TSH-R proteins in the membrane of thyrocyte are quite stable and the receptor signaling in the thyrocyte’s membrane is controlled mainly through TSH levels circulating in thyroid tissue [107]. 

This thyroid-specific TSH-R molecule was found to be involved in the pathogenesis of thyroid diseases including thyroid cancer [107,108,109]. Research suggested that activation of TSH-R may play a pro-oncogenic and growth-promoting role in differentiated thyroid cancer [110,111,112]. In turn, TSH, acting through its receptor, has the potential of stimulating the growth of differentiated thyroid cancer [113]. Under the TSH suppressive condition, a poorer outcome in patients was found to be strongly related to low expression of TSH-R gene in thyroid tissue [114]. Moreover, the studies of properties of this receptor in normal and pathological human thyroid tissues revealed that the number of binding sites was reduced in the pathological tissues [115], that levels of TSH-R gene expression were significantly lower in carcinoma tissues than in normal tissues [109,116], that expression of TSH-R was persistently maintained and, sometimes, TSH-R was hyperactivated in differentiated thyroid tissues and tumors, but this expression was lost in undifferentiated thyroid cancer [117,118], etc. Therefore, it was hypothesized that TSH-R can serve as one of markers of thyroid differentiation [119]. Moreover, based on the observation that most thyroid cancers still express the TSH-R [116], its messenger RiboNucleic Acid (mRNA) has been used as a highly sensitive and specific marker for detecting thyroid cancer cells in peripheral blood [120,121]. 

Overall, it was concluded that the levels of expression of TSH-R gene and its mRNA might have an important diagnostic value [114,115,116,117,118,119,120,121]. In particular, TSH receptor might be persistently expressed in all differentiated thyroid tissues and tumors but lost in undifferentiated carcinomas [118].

Moreover, TSH-R expression might play an important role in clarifying the onset, evolution, and results of therapy of thyroid cancer [117]. As it was mentioned in the first section of Results, TSH suppression was implicated into postoperative management of thyroid function in differentiated thyroid cancer [91,92,93,94,95]. Additionally, it was shown that thyroid cancer cells in primary culture respond to TSH stimulation by activating the cyclic-AMP cascade that promotes cell growth [122,123]. In contrast, expression of the TSH-R is markedly decreased in poorly differentiated thyroid cancers [119,124,125]. However, TSH responsiveness could render some thyroid cancer cells less susceptible to manipulation of TSH concentrations [126,127]. 

Overall, stimulation of TSH production can predict enhanced growth of well-differentiated thyroid cancer cells [122,123,124,125].

### 2.4. Remaining Questions of Studies of TSH, TSH-R, and Clock Genes in Thyroid Carcinoma

Considering the practical importance of uncovering the potential preoperative biomarkers for thyroid carcinoma, it is necessary to emphasize that the levels of TSH-R expression (see Section 2.3) might be associated with the circadian clockwork (see Section 2.2) and the levels of TSH in thyroid tissue (see Section 2.1). For instance, the circadian clocks might determine or, conversely, might be dependent upon the regulation of TSH-R responses to the administration of different concentrations of TSH and other thyroid hormones [2]. Therefore, it is reasonable to expect to find a significant link between thyroid cancer and abnormalities of diurnal profile and mean daily levels of activation of TSH-R. However, there were no studies designed to evaluate the responses of TSH-R to administering TSH or recombinant human TSH (rhTSH) into tissues containing of benign or malignant thyrocytes. This gap contrasts with the results of intensive research on TSH levels in serum [73,74,75,76,77,78,79,80,81,82,83,84,85,86,89,90,91], circadian clock genes expression [2,39,64,70,103,104,105] in thyrocytes, and TSH-R gene activation at thyrocytes’ membrane [107,108,109,110,111,112,114,115,116,117,118,119] (Table 1). 

Another underexplored issue in such research is testing the effects of TSH simulation on expression of various circadian clock genes in thyroid cancer. Moreover, the circadian characteristics of responses to such simulation remains to be explored. Finally, the diurnal profiles of circadian clock genes’ expression in benign or malignant thyrocytes require further investigation (Table 1). For instance, the circadian rhythmicity in cultured human primary thyrocytes from benign and malignant tissue was described only in one of reviewed study [39]. 

Overall, the evaluation of research findings in the previous three sections of Results reveled a lack of studies addressing interrelationships between TSH, its receptor, and circadian clock genes in patients with indeterminate thyroid cytology. 

Therefore, a systematic search of the PubMed database was performed to identify publications that might be relevant to the exploration of these interrelationships. Only three publications remained eligible after applying the ineligibility criteria for initially identified publications (see their description and other details of this search in Table S1). The search confirmed that addressing the interrelationships between TSH, its receptor, and circadian clock genes remained unexplored issue of the studies involving patients with thyroid cancer.

## 3. Discussion

Here, the findings of studies of the links of thyroid cancer to the circadian clockwork dysfunctions and abnormal activities of TSH and its receptor were evaluated. They suggested a plausibility of such links. However, it was also found that studies aimed on exploration of the interrelationships between these dysfunctions and abnormal activities in patients with indeterminate thyroid cytology are lacking. These unexplored issues might be addressed in future studies aimed on detecting, in the framework of this single study, the abnormalities in diurnal profiles of expression of circadian clock genes and TSH-R and the abnormalities in responses of these profiles to administration of TSH or rhTSH (Table 1). For such a combined study of TSH-R and circadian click gene expression, in vitro experiments with cultured benign and malignant thyrocytes might be performed in a similar way to that applied in the Mannic et al. study [39]. Thus far, the samples of cultured normal and malignant thyrocytes have not been examined in in vitro experiments designed to determine whether the manipulations with the levels of TSH or rhTSH administered to thyroid tissue can lead to changes in the diurnal profile of activation of this receptor in parallel with some other changes, e.g., in levels and diurnal profiles of expression of several circadian clock genes (Table 1).

Results of the proposed studies might answer some important questions such as: are benign and malignant thyrocytes different not only in levels and diurnal profiles of expression of circadian clock genes, but also in the diurnal profiles of responses of TSH-R to TSH administration to thyroid tissue? Are the circadian clockwork abnormalities linked to up- or down-regulation of expression of TSH receptor? Can the changes in the levels of administered TSH change the mean levels and diurnal profiles of expression of TSH-R and circadian clock genes? Can these changes in diurnal profiles of expression of TSH-R and circadian clock genes be interrelated? The accounting for circadian rhythmicity of expressions of TSH-R and clock genes can span the three directions of a search for potential preoperative biomarkers for thyroid carcinoma including and provide evidence for diagnostic value of simultaneous examination of TSH, TSH-R, and circadian clock genes (Table 1).

However, it has to be noted that performing the proposed study might be delayed due to the necessity to apply a more complex (and costly) experimental procedure in comparison to the procedures previously applied in the studies of serum TSH concentrations and expression of TSH-R. For instance, it would be necessary to cultivate many samples for many days including, at least, a three-day interval of multiple measurements (at different clock times) of the effects of varying (e.g., normal, and elevated) TSH or rhTSH levels on the diurnal profiles of expression of this receptor and several circadian clock genes. Nevertheless, now we are witnessing a very rapid development of molecular-genetics research technology that gives us hope that, in the near future, the cost-efficient methods for the screening of a large number of samples will be available for the proposed study. In particular, such research would require multiple testing on a 24-h interval, but such possibility has been permitted by the recent development of the methodologies for the diagnosis of thyroid cancer. Thyroid tissue might be cultured for many days, and, in the absence of such tissue, liquid biopsy can be repeatedly performed at different clock times in a noninvasive way.

## 4. Materials and Methods

A systematic search of the PubMed database was performed to identify the publications relevant to several associations that remained unexplored in the studies involving patients with thyroid cancer. These are the associations of circadian disruptions with (1) serum TSH levels, (2) TSH suppression, (3) TSH stimulation, (4) expression of TSH-R, and the associations of TSH-R expression with (5) TSH levels and TSH suppression, (6) TSH levels and TSH stimulation, and (7) TSH suppression and TSH stimulation. 

The search strategy included the applying several ineligibility criteria for initially identified publications: (1) patients with other diseases, (2) less than 6 patients, (3) non- patients, (4) measurements of serum TSH or expression of TSH-R are not reported, (5) the interval of measurement of serum TSH is limited to night and/or morning subintervals of the 24-h cycle, (6) review, and (7) not in English. The results of this search are briefly reported in Results and described in more details in Table S1.

## 5. Conclusions

The preoperative examination of samples of thyroid tissue obtained in a search for preoperative markers for thyroid carcinoma plays a critical role in diagnostic of this disease and in avoiding unnecessary surgeries and treatment of asymptomatic cancerous TN. However, postoperatively, 70–90% of thyroid FNA cases are found to be benign. Therefore, preoperative markers for thyroid carcinoma play a critical role for the attempts to avoid unnecessary surgery and delays with the treatment of this disease. Evaluation of results of earlier and more recent studies provided evidence for the links of thyroid cancer to the circadian clockwork dysfunctions and abnormal activity of TSH and TSH-R. The links of thyroid cancer to the properties of TSH-R and circadian clocks genes might be further clarified in in vitro experiments on cultured benign and malignant thyrocytes. The design of these experiments might require the simultaneous testing the diurnal profiles of expression of TSH-R and circadian clock genes under both normal and challenged TSH levels. These experiments would also require multiple testing throughout the day, but the recently developed methodologies for the diagnosis of thyroid cancer permits a possibility of such testing.

## Figures and Tables

**Table 1:** Evidence for potential clock-associated preoperative biomarkers for thyroid carcinoma.

Marker	Was Evidence Provided?	References
TSH concentration in serum		
High or low levels of marker	It was provided	[73,74,75,76,77,78,79,80,81,82,83,84,85,86,89,90,91]
Its circadian rhythmicity	It was insufficient	[128]
Circadian clock genes’ expression in thyrocytes		
High or low levels of marker	It was provided	[2,39,64,70,103,104,105]
Its circadian rhythmicity	One study provided	[39]
TSH-R gene’s expression in thyrocytes		
High or low levels of marker	It was provided	[107,108,109,110,111,112,114,115,116,117,118,119]
Its circadian rhythmicity	Remains to be provided	-
TSH-R protein response to TSH or rhTSH levels in thyroid tissue		
High or low levels of marker	Remains to be provided	-
Its circadian rhythmicity	Remains to be provided	-
TSH-R & Circadian clock genes’ expression in thyrocytes		
High or low levels of marker	Remains to be provided	-
Its circadian rhythmicity	Remains to be provided	-

Notes: TSH: Thyroid-Stimulating Hormone or thyrotropin; rhTSH: recombinant human TSH; *TSH-R*: TSH receptor gene; Circadian clock genes: a set of genes (including *BMAL1* and *CRY2*) involved in a transcriptional-translational feedback loop regulating the circadian clocks in the vast majority of mammalian cells; *TSH-R* & Circadian clock genes: A proposed study involving multiple measurements of expression of *TSH-R* and several Circadian clock genes (*BMAL1*, *CRY2*, etc.).

## Data Availability

Not applicable.

## Supplementary Materials

The following supporting information can be downloaded at: https://www.mdpi.com/article/10.3390/ijms232012208/s1. References [111,114,127,128] are cited in the supplementary materials.
