# Supplementary material for: Prospects of Testing Diurnal Profiles of Expressions of TSH-R and Circadian Clock Genes in Thyrocytes for Identification of Preoperative Biomarkers for Thyroid Carcinoma
Source: Int J Mol Sci. 2022 Oct 13;23(20):12208. doi: 10.3390/ijms232012208 (PMC9603503; doi:10.3390/ijms232012208)
Supplement: Supplementary file 1 [file ijms-23-12208-s001.zip › ijms-1956262-supplementary.pdf]

Table S1 Combinations of obligatorily and additionally included terms and the number of identified publications.

to

"Prospects of testing diurnal profiles of expressions of TSH-R and circadian clock genes in thyrocytes for identification of preoperative biomarkers for thyroid carcinoma"

Details of the Literature Search (see also 4. Materials and Methods)

Table S1. Combinations of obligatorily and additionally included terms and the number of identified publications.

Obligatory	(thyroid cancer or thyroid carcinoma or thyroid nodule) and (patients)						Identified publications	
Additional	TSH	TSH suppression	TSH stimulation	circadian	TSH-R	gene expression	Initially	Eligible
1	+	-	-	+	-	-	25	1
2	-	+	-	+	-	-	5	0
3	-	-	+	+	-	-	9	0
4	-	-	-	+	+	-	0	0
5	+	+	-	-	+	+	1	1
6	+	-	+	-	+	+	5	1
7	-	+	+	-	+	+	0	0
In total, the results of 7 searches, with or without duplicated references from other combinations of terms (1-3,5,6)								
with							45	3
without							30	3

Notes. Obligatory: the list of terms included in any of combinations; Additional: two or four terms were added to the always included terms (denoted as "+"). Initially and Eligible: Number of initially identified and eligible publications. See the details on three eligible and 27 ineligible references below.

Results Reported in Three Eligible Publications

Combination of terms 1. A search for literature on the circadian rhythm of TSH in patients with thyroid carcinoma.

(ref 128. Custro N, Scafidi V, Notarbartolo A. Alterations in circadian rhythm of serum thyrotropin in critically ill patients. Acta Endocrinol (Copenh). 1992 Jul;127(1):18-22).

Serum concentrations of thyrotropin (TSH) were measured in blood samples collected every 2 h for 24 h from nine patients (six with malignancy, two with liver cirrhosis, one with chronic renal failure), who had subnormal levels of both triiodothyronine (T3) and thyroxine (T4), in the absence of history, symptoms or signs of thyroid disease. the mean level (mesor) in the rhythm of the patients was found to be significantly lower than that of healthy subjects (0.96 vs 2.18 mU/l); the amplitude of rhythmical daily variations also was lower in patients than in healthy subjects (0.23 vs 0.56 mU/l), even though the amplitude/mesor ratio was similar (23% vs 26%). Lastly, the highest level in the TSH rhythm of the patients was found to be in the late afternoon, in contrast to healthy subjects, who had a TSH surge after midnight.

However, the question remains about such disturbances of the circadian TSH rhythm in patients with thyroid cancer but without symptoms or signs of thyroid disease.

Combination of terms 5. A search for literature on the expression of TSH receptor (TSH-R) under the TSH suppressive condition.

(ref 114. Tanaka K, Sonoo H, Yamamoto Y, Udagawa K, Kunisue H, Arime I, Yamamoto S, Kurebayashi J, Shimozuma K. Changes of expression level of the differentiation markers in papillary thyroid carcinoma under thyrotropin suppression therapy in vivo immunohistochemical detection of thyroglobulin, thyroid peroxidase, and thyrotropin receptor. J Surg Oncol. 2000 Oct;75(2):108-16. doi: 10.1002/1096-9098(200010)75:2<108::aid-jso7>3.0.co;2-v).

Differences in the expression levels of Thyroglobulin (Tg), Thyroid peroxidase (TPO) and TSH-R in primary and recurrent specimens under a suppressive serum TSH condition were elucidated in 26 papillary carcinoma patients. Under the TSH suppressive condition, low expression of TSH-R in the recurrent tissue was strongly related to a poorer outcome in the patients.

Combination of terms 6. A search for literature on the expression of TSH receptor (TSH-R) under the TSH stimulating condition.

(ref 127). Rosignolo F, Maggisano V, Sponziello M, Celano M, Di Gioia CR, D'Agostino M, Giacomelli L, Verrienti A, Dima M, Pecce V, Durante C. Reduced expression of THRβ in papillary thyroid carcinomas: relationship with BRAF mutation, aggressiveness and miR expression. J Endocrinol Invest. 2015 Dec;38(12):1283-9. doi: 10.1007/s40618-015-0309-4).

Thirty-six patientd with human papillary thyroid carcinomas (PTCs) were divided into two groups according to the 2009 American Thyroid Association risk classification (17 low, 19 intermediate). A minor reduction of TSH-R expression was reported, but it was concluded that no significant deregulation of TSH-R was found and that this result confirmed their previous finding regarding of the close to normal levels of TSH-R in PTCs (ref 111. D'Agostino M, Sponziello M, Puppin C, Celano M, Maggisano V, Baldan F, Biffoni M, Bulotta S, Durante C, Filetti S, Damante G, Russo D. Different expression of TSH receptor and NIS genes in thyroid cancer: role of epigenetics. J Mol Endocrinol. 2014 Jan 30;52(2):121-31. doi: 10.1530/JME-13-0160.)

Reasons for Ineligibility of 43 Excluded Publications

Combination of terms 1. A search for literature on the circadian rhythm of serum TSH in patients with thyroid carcinoma.

Other disease – 14, not patients – 1, absence of measurements – 1, only night and/or morning measurements – 4, review – 2, not in English – 2. In total, 24.

Combination of terms 2. A search for literature on the circadian rhythm and TSH suppression in patients with thyroid carcinoma.

Other disease – 1, less than 6 patients – 1, absence of measurements – 1, only night and/or morning measurements – 1, review – 1. In total, 5.

Combination of terms 3. A search for literature on the circadian rhythm and TSH stimulaion in patients with thyroid carcinoma.

Other disease – 7, only morning and night measurements – 2. In total, 9.

Combination of terms 6. A search for literature on the expression of TSH receptor (TSH-R) under the TSH stimulating condition.

Other disease – 4. In total, 4.
